# From data collection to action: environmental health information management practices in eleven municipalities in the east coast of South Africa

**DOI:** 10.3389/frhs.2026.1890673

**Published:** 2026-08-18

**Authors:** Siphesihle Siyamukela Masimula, Mpinane Flory Senekane, Nisha Naicker

**Affiliations:** 1 Department of Environmental Health, Faculty of Applied and Health Sciences, Mangosuthu University of Technology, Umlazi, South Africa; 2 Department of Environmental Health, Faculty of Health Sciences, University of Johannesburg, Johannesburg, South Africa; 3 National Institute for Occupational Health, National Health Laboratory Services, Johannesburg, South Africa

**Keywords:** decision-making, environmental health, environmental health information management, environmental health services, public health, South Africa

## Abstract

**Background:**

The availability of reliable information in the delivery of environmental health services (EHS) is crucial to identifying environmental health threats in communities and deploying targeted interventions. This requires environmental health information management practices and systems to be evaluated from time-to-time to bring efficiency and enable continuous improvement for improved public health outcomes. As a result, this study assesses environmental health information management in municipalities that provide EHS in the east coast of South Africa.

**Methods:**

A descriptive quantitative cross-sectional study design was employed, combining an online survey of 105 environmental health practitioners (EHPs) and document reviews across 11 municipalities. Descriptive and inferential statistical analyses were conducted through IBM SPSS Statistics.

**Results:**

Findings highlight over-reliance on paper-based data management processes, resulting to data duplication (88.6%) and increased workload (90.5%) for EHPs (*p* = 0.004). Inadequate training, lack of adequate digital tools (17.1%), inconsistent support (61.9%) and poor feedback (46.6%) impeded effective information utilisation for public health action. The study further underscored the need for improved quality assurance, feedback mechanisms and technical support. Governance, organisational capacity, limited digital infrastructure, and insufficient training emerged as key barriers.

**Conclusion:**

Substantial environmental health information management strengthening through digitalisation, improved governance, capacity-building, and stronger quality assurance systems are required. A comprehensive and integrated digital EHIS is needed to improve data quality, information utilisation, and evidence-based environmental health decision-making in South Africa.

## 1 Introduction

Sound and reliable information is the cornerstone of all the components of the health system for insight-driven decision-making (1). Hence, reliable information is crucial for guiding and improving policies, governance, research, resource allocation, service delivery, and human resource capacity building (2). Therefore, data collection provides a foundation for routine health information systems (RHIS) to be able to generate intended information during the routine provision of health services. RHISs are used by different countries around the globe to regularly collect data to meet prescribed health information demands, offering a quick overview of the state of public health and required interventions (3, 4). The RHISs are also referred to as health facility-based or community health information systems, which are specific to rendered health services (3).

In the provision of Environmental Health Services (EHS) to identify, prevent, reduce and control environmental threats to human health, the availability of sound and reliable information is key to allowing health practitioners to develop environmental health situational analyses, monitor trends and patterns, and implement data-informed interventions (5, 6). EHS, among many programs, involves environmental pollution control, chemical safety and the control of hazardous substances, climate change and health, waste management and general hygiene monitoring, food safety and hygiene, prevention and control of communicable diseases, water quality and safety, vector control, health surveillance of premises and the management of human remains (7–9). In the agenda to pursue environments that are conducive to human health, different countries around the world have environmental health information systems (EHISs) to engage in environmental health monitoring and intelligence to better understand the relationship between environmental hazards and public health for a better response (10–13).

In South Africa, an EHIS exists within the District Health Information System (DHIS), which is a broader health information system that harbours almost all public health services in South Africa (14). This EHIS is made up of environmental health indicators to monitor the state of the environment in connection with the environmental burden of disease in the country (15). Therefore, environmental health practitioners (EHPs) collect and report data to this system, as they provide routine EHS in districts and metropolitan municipalities, port health services at Ports of Entry and provincial EHS within different provincial departments of health (14, 15). The majority of EHS programs in South Africa are provided by municipalities (7). Owing to their close proximity to communities, municipalities are strategically well-positioned to identify and address environmental health risks at the local level (16, 17).

At the centre of environmental health surveillance and monitoring is data management and information utilisation to provide evidence and inform decision-making (18, 19). However, environmental health information management was identified as a challenge in South Africa because of the inability to cover comprehensive environmental health issues, spot trends of exposure and enable relevant interventions to be enacted (18, 20, 21). A lack of data-driven approaches has also been recognised as hindering the development and effectiveness of EHS in South Africa (21). This finding corroborated the findings of other studies, which revealed inefficiencies in environmental health information management and suggested that reforms are needed in this regard (22–25). Weak and fragmented information management processes that are unable to strengthen and support health systems have been reported as part of the challenges in low-to-middle-income countries (4, 26–32). This calls for RHISs in middle to low-income countries to be evaluated from time to time and remodelled to ensure they are relevant to the current times and public health challenges (33). As EHS are part of the broader public health system, the RHIS that is utilised in the provision of these services must be assessed (13, 18, 34). In response to the need for reforms in environmental health information management, as revealed by the literature, an opportunity arose to contribute to the body of knowledge and practice on the status quo in the provision of EHS. A number of researchers have called for more studies in environmental health information management in South Africa (18, 22, 24, 25). This indicates a research gap, which this study is also contributing towards addressing by providing empirical evidence and establishing a baseline understanding.

**Figure 1:**
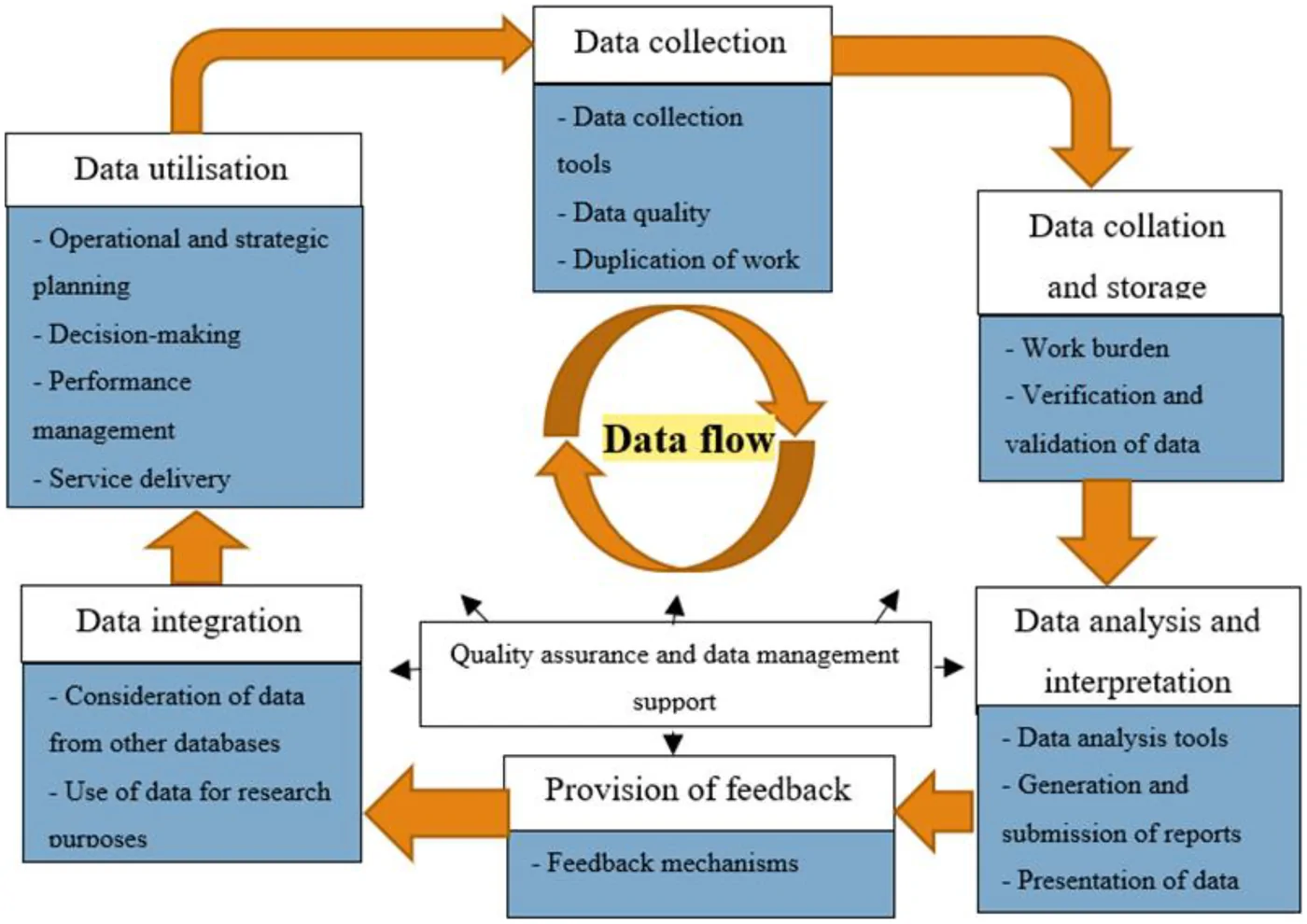
Conceptual framework for this study.

As a result of this background, this study was conducted to assess environmental health information management in municipalities that provide EHS in the province of KwaZulu-Natal, in South Africa. An assessment of an RHIS provides a platform for interventions to be developed to improve its performance, present new opportunities and maximise best practices (3, 13, 34). This assessment was grounded on the conceptual framework that was developed for this study, which is depicted in Figure 1.

This conceptual framework steered this study by pointing out key data management elements and support pillars in the data flow process, as per the guidance of the Updated DeLone and McLean Information Systems Success Model, the Performance of Routine Information System Management (PRISM) Framework, and the Information Cycle Framework (35–38). Therefore, this study draws on and integrates complementary theoretical perspectives from the above-mentioned established health information system frameworks. These frameworks complement each other, although they have different systematic approaches towards evaluating RHISs (39). As a result, they were synthesised by aligning their distinct conceptual emphases into a unified structure for assessing environmental health information management. In the evaluation of routine health information management, this is done to integrate factors of interest into a single framework to allow a detailed assessment that suits an information system of interest (26). The PRISM Framework states that the performance of RHISs relies on data quality and continuous data utilisation, among other factors (36). Therefore, in this study, the assessment was based on users’ training and their perceptions of the EHIS, data quality, data safety, provision of feedback, data integration, and data utilisation. Quality assurance and data management support were also assessed in relation to the whole data flow steps in the information cycle. These two support pillars are important activities in ensuring that an RHIS functions effectively (40).

The term information cycle refers to the stages involved in the handling of data as it is distributed over time to end up as usable information (35). Heywood and Rhode (2001) conceptualised an Information Cycle Framework, which shows how data are managed and applied to ensure the timely generation of relevant and useful information (35, 41). Therefore, the Information Cycle Framework outlines the links between different stages of the information cycle, from data collection to processing, analysis, presentation, interpretation, and utilisation (35). The data collection stage involves the collection of essential data sets that are available based on the health services provided and data demands. Once the data are collected, collation takes place for processing, where vast amounts of data are combined into a format that can be analysed. Data are then analysed into indicators that are presented through graphs, tables, maps, figures, narratives, etc. Data analysis enables the interpretation of data, thereby creating information that can be used for decision-making and action (30). The assessment in this study followed the flow of data in the information cycle, as highlighted above. The concepts of interest in this study included quality assurance and data management support. Based on the Updated DeLone and McLean Information Systems Success Model, quality assurance and data management support that is provided to users (including training, technical support and the availability of resources) improves an information system's performance (37). The PRISM Framework supports this view, as it emphasises that organisational and technical factors directly influence the behavioural factors associated with users (36). These factors are critical when it comes to applying RHIS processes to strengthen the delivery of health services and ensure continuous improvement. In this assessment, the Information Cycle Framework provided a process-oriented approach through sequential data flow stages. The PRISM Framework informed the inclusion of key determinants of a functional RHIS. The DeLone and McLean Model contributed a focus on system support and quality dimensions, particularly on aspects such as user support, training, and resource availability, which are critical for the effectiveness of an information system. The integration of these frameworks enabled the study to assess functional processes of data management, as well as key factors that influence the success of a system.

## 2 Materials and methods

### 2.1 Study setting

**Figure 2:**
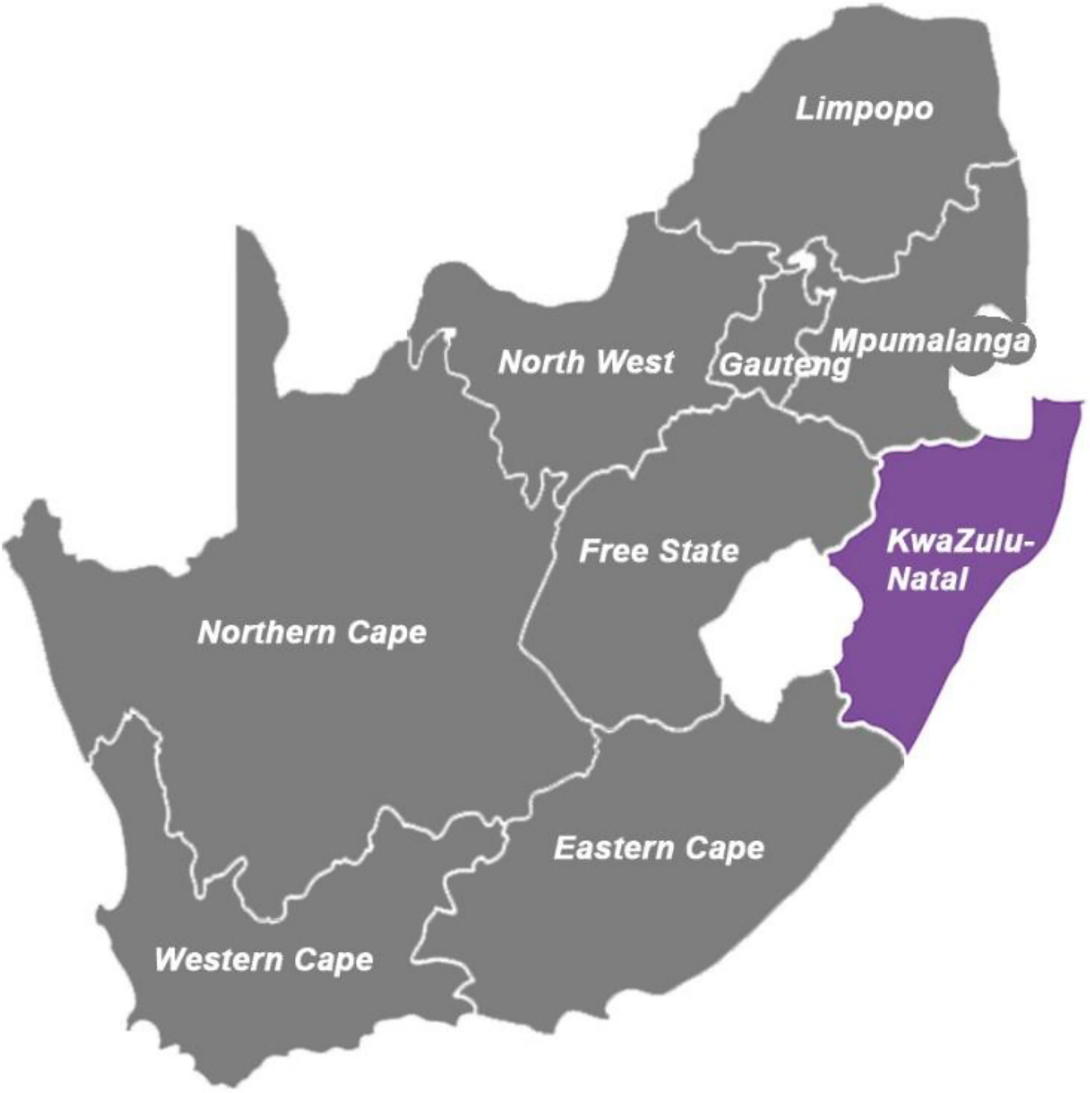
Map of South Africa, showing Kwazulu-Natal Province (source: grain SA, 2024) (79).

This study was conducted in 2024 in the province of KwaZulu-Natal, located on the east coast of South Africa. As shown in Figure 2, this province is one of the nine administrative divisions of South Africa. It is the second-largest provincial economy after Gauteng province in the country (42). The province has a population of approximately 12.4 million people, which makes it the second most populated province after Gauteng (42). Thirteen municipalities (one metropolitan municipality, 10 district municipalities and two local municipalities) provide EHS in this province (43). All 13 municipalities were requested to form part of the study.

The provision of EHS in the KwaZulu-Natal municipalities was reported to be affected by budgetary constraints, a lack of human resources, a lack of technical tools for trade and less prioritisation in terms of service delivery improvements (43). These issues can directly and indirectly affect environmental health information management and consequently affect the surveillance and analytical capacity of EHS for information dissemination and utilisation. These issues were reported while the province and the country were experiencing extreme weather patterns from climate change, which posed threats to public health (44, 45). Other environmental health issues in the KwaZulu-Natal province included poor air quality in industrialised areas, water scarcity, water quality issues, infectious diseases, the proliferation of vectors, food safety and hygiene compliance issues, poor waste management practices, rapid urbanisation and disregard of the law by informal business premises (23, 46–50).

### 2.2 Study design

A descriptive quantitative cross-sectional study design was employed to conduct this study, using an online survey for primary data and a document review process for secondary data. This multi-source descriptive approach allowed the document review to complement the online survey by enabling triangulation, while enhancing the depth and validity of the study. The chosen study design enabled the researchers to map population characteristics and generate a holistic snapshot of practices, systems, and institutional arrangements that underpin environmental health information management at a defined point in time, in a cost-effective and time-efficient manner. This was approach was appropriate since environmental health information management systems and practices are context-dependent and operationally embedded (51). Furthermore, given the limited empirical evidence in this area, the chosen study design was identified as well-suited to establish baseline knowledge on how data are collected, managed, stored, and used within the system while exploring challenges and best practices, thereby supporting future research, system strengthening, and policy development.

### 2.3 Study population

The study population for the online survey included 241 EHPs who were employed in the 13 municipalities, stationed at an operational level of EHS, and registered with the Health Professions Council of South Africa under the Environmental Health Board as independent practitioners. The total number and database of EHPs were obtained from the South African Local Government Association and the management of the respective municipalities (52). For the document review, the study population included 13 municipalities. However, of the 13 municipalities, two did not give permission to form part of the study. Reasons were not stated. As a result, the population of municipalities decreased to 11, and that of EHPs decreased to 228.

### 2.4 Sampling method

From a population of 228 EHPs, the minimum sample size was calculated as 68, based on a 95% confidence level, a 10% margin of error and an assumed proportion of 50% (*p* = 0.5). The assumption of 50% was used in the absence of prior estimates. Given the relatively small population, a finite population correction was applied in Epi Info Version 7.2.6.0 (Supplementary Material 1). The calculation further assumed simple random sampling. Even though the minimum sample size was set, a census approach was attempted by inviting all 228 eligible EHPs via email to form part of the study. However, participation was ultimately determined by access to the recruitment email (with a consent form and a link to access the online questionnaire) as well as availability and willingness to respond, resulting in a sample of 105 EHPs. A convenience sampling method was therefore applied in practice. This method was considered appropriate due to the geographical dispersion of the study area and recruits (EHPs) being scattered in terms of the location of their municipalities and workstations (offices), which made probabilistic sampling logistically challenging. The email addresses of EHPs were received from all the participating municipalities, and the recruitment email was sent to all municipalities sequentially. However, access or receipt of the message could not be guaranteed, which further constrained the feasibility of random or systematic sampling. This made the convenient sampling method suitable for this study. Additionally, online surveys among health professionals are often associated with low response rates (53). As a result, inviting the entire population and using the convenient sampling approach was a strategy to counter the anticipated low response rate, and maximise participation. Overall, this approach ensured that the achieved sample size exceeded the minimum required, thereby improving the precision of estimates, while remaining feasible, efficient, and cost-effective. For the document reviews, no sampling of municipalities was conducted.

### 2.5 Data collection

#### 2.5.1 Online survey

To collect data from EHPs, the Google Forms application was used to develop an online self-administered questionnaire (Supplementary Material 2). Thereafter, an email with the study's information letter and a web-based link to access the informed consent and the questionnaire was sent to all the EHPs. The questionnaire was mainly made up of closed-ended questions with preset responses in the form of multiple choices, checkboxes, yes or no and Likert scales. It was developed to collect data on demographics, the training of EHPs, data collection, storage, collation and reporting; perceptions of the EHIS; quality assurance and data management support; data analysis and provision of feedback; collaborations for data integration and information utilisation. The abovementioned constructs that led this study were derived from the conceptual framework depicted in Figure 1. The online survey was open for participation from the beginning of January 2024 to the end of February 2024, after which no more responses were coming, and the desired minimum sample size was exceeded. The online survey and its questionnaire were developed and enrolled for the purpose of this study. The questionnaire and its content were reviewed and validated by experts, and pre-tested to strengthen its reliability, validity, and feasibility. The use of an online survey facilitated efficient and cost-effective data collection.

#### 2.5.2 Document review

In the document review process, secondary data were extracted from the documents of the municipalities through a data extraction sheet (Supplementary Material 3), which was also reviewed, validated by experts and pre-tested. This instrument was designed to guide the documents to be focused on, as well as the targeted content to be reviewed to enable observations, data extraction and consolidation. The documents of focus included guidelines, standard operating procedures, environmental health data management tools, reports, minutes of meetings, and operational and strategic plans. In this case, the document review played a critical role in availing evidence and assessing practices as well as the formal and documented dimensions of the system. This was imperative to enable researchers to identify alignments or discrepancies between the self-reported results and documented systems or recorded practices, which is important in assessing system functionality and implementation gaps. The document review process to extract data was conducted in the same period when the survey was open for participation, using an approach of one municipality at a time. In all 11 municipalities, the researcher made appointments with their EHS departmental managers and visited their central (headquarters) offices to peruse and review their various institutional documents and information system(s). The data extraction sheet was created in an online form via the Google Forms application, to enable seamless data collection. Therefore, during the perusal and review of working documents and systems in the municipalities, data were fed to the online data extraction sheet, and where necessary, brief notes were made by the researcher.

### 2.6 Data analysis

The responses received from the online survey and the data captured during the document reviews were automatically delivered to Google Sheets as two separate spreadsheets, which were then saved in Microsoft Excel and data cleaning was conducted. The spreadsheet from the survey (Supplementary Material 4) was imported into IBM SPSS Statistics 29.0 for data analysis. In this case, descriptive and inferential statistics were used to analyse the data. Inferential statistical analysis was performed to measure associations between selected variables. As a result, cross-tabulations were conducted on 2-by-2 tables, and Fisher's exact tests were used to determine the *p-values*. For statistically significant associations, the Phi coefficient was used to indicate the strength of those associations. When the Phi coefficient value was less than 0.3, it indicated a weaker association; when it was between 0.3 and 0.5, it showed a medium association; and when it was greater than 0.5, it indicated a stronger association (54). As part of inferential statistics, nonparametric (Spearman's Rho) correlation tests were also conducted, as the data were not normally distributed. The *p-value* was used to measure the significance of the correlation. The correlation coefficient value (Rho) was used to measure the strength of the correlation between the variables. A correlation coefficient value that was closer to one, from 0.50, denoted a stronger correlation (55). A correlation coefficient between 0.30 and 0.49 indicated a medium correlation, and a coefficient between 0.10 and 0.29 indicated a weaker correlation. Furthermore, a negative correlation coefficient value indicated an inverse (negative) correlation between the variables. The second spreadsheet from the document review was analysed in Excel by the researcher. Descriptive statistics were used to calculate the frequencies of the responses and the percentages of the data extraction dataset.

### 2.7 Ethical aspects

This study received ethical approval from the Faculty of Health Science's Research Ethics Committee (REC-2469-2023) at the University of Johannesburg. Gatekeeper's permission was granted by both the South African Local Government Association and the management of the municipalities that participated. Every participant gave their informed consent to participate voluntarily. The right to withdraw from the study at any moment was explained to the respondents. Respondents received dignified and respectful treatment, and there was no harm that took place. Confidentiality, privacy, and anonymity were guaranteed.

## 3 Results

The results of this study are presented in this section with the consideration of the data flow process, key data management elements and support pillars as per the conceptual framework of this study and its objective.

### 3.1 Demographics

Table 1 shows the demographic characteristics of the respondents who participated in the online survey.

**Table 1:** Demographic characteristics of the respondents.

	Demographic variables	Frequency	Percentage (%)
Gender	Male	46	43.8%
	Female	59	56.2%
Age group	20 to 30 years	11	10.5%
	31 to 40 years	68	64.8%
	41 to 50 years	21	20%
	51 to 65 years	5	4.9%
Years of experience	Less than 5 years	11	10.5%
	6 to 10 years	45	42.9%
	11 to 20 years	42	40%
	21 years and above	7	6.7%
Computer literacy	Basic level	12	11.4%
	Intermediate level	55	52.4%
	Advanced level	38	36.2%
Digital literacy	Basic level	10	9.5%
	Intermediate level	46	43.8%
	Advanced level	49	46.7%
Total number of respondents = 105			

### 3.2 Perceptions of environmental health practitioners

On perceptions in relation to the EHIS and data management, as depicted in Table 2, the majority of the respondents (*n* = 99; 94%) valued the importance and usefulness (*n* = 93; 89%) of the data they fed to the system. As they recognised the importance of their data, they also saw its usefulness (*p* < 0.001; Rho=0.503). This indicates a statistically significant and moderate-to-strong positive correlation, demonstrating that most respondents valued and understood the significance and usability value of their data and the system.

**Table 2:** Results on the rating of the importance and usefulness of the data fed to the EHIS by EHPs (*n* = 105).

Variables		Rating scale on the importance and usefulness of data and the results in numbers and percentages (%)				
Rating of the importance of data by the respondents	Rating scale	Not Important	Slightly important	Moderately important	Important	Very important
	Counts (%)	1 (1%)	5 (4.8%)	11 (10.5%)	31 (29.5%)	57 (54.3%)
Rating of the usefulness of data by the respondents	Rating scale	Not useful	Slightly useful	Moderately useful	Useful	Very useful
	Counts (%)	5 (4.8%)	7 (6.7%)	15 (14.3%)	52 (49.5%)	26 (24.8%)
Total number of respondents = 105						

In contrast to the above positive perception, 90.5% (*n* = 95) of the respondents felt burdened by data collection in their line of duty, but to different extents, as indicated in Table 2. This demonstrates the existence of a perceived increased work burden due to data collection among many EHPs.

### 3.3 Environmental health data collection

When it comes to data collection, the results revealed that EHPs in their routine work generate data and record them in checklists (*n* = 87; 82.8%), reports (*n* = 77; 73.3%), daily/weekly activity books (*n* = 67; 63.8%), and notebooks and diaries (*n* = 53; 50.4%). These documents serve as data sources for the data that are captured in weekly and monthly input forms, which serve as data collection tools for reporting and recording data into the EHIS. On this basis, it was evident that the studied municipalities used manual paper-based systems to collect and record data. On the availability of working tools, the respondents indicated having access to pens and pencils (*n* = 96; 91.4%), papers (*n* = 87; 82.8%), computers (*n* = 88; 83.8%), printers (*n* = 86; 81.9%), scanners (*n* = 80; 76.1%) and the internet (*n* = 84; 80%). However, there was limited access to technical tools (*n* = 18; 17.1%) and handheld devices (*n* = 16; 15.2%), especially those that are technologically advanced to be able to automate manual processes and improve productivity.

Most of the EHPs (*n* = 93; 88.6%) indicated that they experienced duplication of work activities and data during data collection and recording. Parallel reporting was experienced frequently by 62.8% (*n* = 66) of the respondents (Table 3). The document review exercise also revealed that data duplication was present in nine municipalities. The existence of data and work duplication indicates elements of redundancy in the data collection tools, as well as the negative effect of parallel reporting. As a result, most of the respondents indicated that data collection tools need to be reviewed to add (*n* = 93; 88.6%) and remove (*n* = 82; 78.1%) some data elements. A moderate positive association (*p* = 0.004; Phi=0.316) was found between the need for the removal of some data elements and experiencing work and data duplication in reporting. This suggests that EHPs who reported duplication of work and reporting requirements were more likely to believe that certain data elements should be removed from the reporting system. However, document reviews also revealed that data elements in the utilised data collection tools were insubstantial in terms of the work of the EHPs, as outlined in the Environmental Health National Norms and Standards, as well as the Environmental Health Scope of Practice. Consequently, few (*n* = 7; 6.7%) of the EHPs were satisfied with the current system. While 25.7% (*n* = 27) were moderately satisfied, 23.8% (*n* = 25) were slightly satisfied, and 43.8% (*n* = 46) were not at all satisfied.

**Table 3:** Results of respondents on selected variables in data management and utilisation (*n* = 105).

Variables	Frequency of the responses presented in numbers and percentages (%)				
	Never	Rarely	Sometimes	Often	Always
Frequency of experiencing parallel reporting (reports requiring similar data).	1 (1%)	16 (15.2%)	22 (21%)	42 (40%)	24 (22.9%)
Participation in the discussion of indicators on the data collection tools.	21 (20%)	46 (43.8%)	26 (24.8%)	10 (9.5%)	2 (1.9%)
Participation in the review of data elements or indicators in the data collection tools.	48 (45.7%)	36 (34.3%)	15 (14.3%)	3 (2.9%)	3 (2.9%)
Frequency of following the prescribed methodologies during data collection.	1 (1%)	8 (7.6%)	17 (16.2%)	54 (51.4%)	25 (23.8%)
Frequency of data verification by supervisors.	13 (12.4%)	23 (21.9%)	33 (31.4%)	24 (22.9%)	12 (11.4%)
Frequency of receiving data management support.	28 (26.7%)	39 (37.1%)	28 (26.7%)	6 (5.7%)	4 (3.8%)
The frequency with which the respondents engaged in data analysis.	8 (7.6%)	19 (18.1%)	30 (28.6%)	36 (34.3%)	12 (11.4%)
The frequency with which the respondents engaged in data integration, by considering data from other databases outside Environmental Health.	12 (11.4%)	25 (23.8%)	38 (36.2%)	25 (23.8%)	5 (4.8%)
Respondent's participation in the use of data to inform, plan and make decisions in their workplaces.	6 (5.7%)	12 (11.4%)	30 (28.6%)	44 (41.9%)	13 (12.4%)
Sentiments and perceptions of the respondents towards their supervisors on how often they utilise the data provided by the EHIS for planning and decision-making.	17 (16.2%)	20 (19%)	42 (40%)	17 (16.2%)	9 (8.6%)
Sentiments and perceptions of the respondents towards their senior management on how often they utilise the data provided on the EHIS for planning and decision-making.	24 (22.9%)	45 (42.9%)	20 (19%)	11 (10.5%)	5 (4.8%)
Total number of respondents = 105					

With respect to the user-friendliness of the data collection tools within the system, the results revealed that most (*n* = 39; 37.1%) of the respondents deemed the data collection tools to be user-friendly at a moderate level, followed by 25.7% (*n* = 27), who selected user-friendly. Very user-friendly was selected by 1.9% (*n* = 2), slightly user-friendly was selected by 16.2% (*n* = 17), and non-user-friendly was selected by 19% (*n* = 20). Statistical tests revealed a moderate negative association (*p* < 0.001; Rho=−0.357) between the user-friendliness of the tools and the work burden on the EHPs. This indicates that the lower the user-friendliness of the tools were, the greater the degree of work burden on the EHPs was reported. It was also found that as the EHPs’ years of age (*p* *=* 0.012; Rho = 0.243) and experience (*p* *=* 0.026; Rho = 0.217) increased, they were slightly more likely to perceive data collection activities as a burden in their work. The weak strength of these two correlations, indicate that age and experience had only limited influence on this perception. However, these results indicate a need for a review of the data collection tools and the entire EHIS to eliminate redundancy and the duplication of data and work and promote data relevancy and completeness.

### 3.4 Data collation and storage

During the document reviews, it was noted that data collation was conducted mainly manually, through a pen, paper and a calculator, or through calculation and recording via a computer. The use of outdated data collation tools, errors in collation, paper-based records and lack of uniformity in the definition of elements were also noted as part of the challenges that negatively affected data quality. The document review further revealed that when the data from EHPs has been collated into a monthly report of an office or subdistrict for further consolidation at the municipal (district) level, they are captured into the Web-DHIS monthly.

To store data and work records, most EHPs indicated that they used paper-based storage mechanisms (e.g., cabinets, files, registers, books, etc.) (*n* = 101; 96.1%) to keep hard copies of records with data and computers (*n* = 71; 67.6%) for soft copies. This implied that the data in file cabinets were vulnerable to tampering, as well as being lost or destroyed. The use of digital systems, such as cloud storage (*n* = 26; 24.7%) and software-based storage (*n* = 9; 8.9%), was very low. During document reviews in four municipalities, different methods of filing or record keeping were applied within the same municipalities, as some offices had centralised storage, whereas others had decentralised the storage of records to EHPs’ offices and desks. A lack of standardisation in record-keeping was noted. Records were piling, and other file cabinets were full; there was a lack of enough space and no concrete strategies for archiving. Five municipalities did not have document management systems or policies to address data storage. Furthermore, in seven municipalities, there was a lack of effective access control measures for paper-based files and registers that were source documents where records and data were captured.

### 3.5 Quality assurance

The results of this study show that discussions on the definitions of indicators and elements were lacking between the EHPs and their managers. This is because 88.5% (*n* = 93) of the EHPs said these discussions took place on occasions that are sometimes and below, as shown in Table 3. The lack of these discussions opens an opportunity for various contradictory interpretations of indicators and elements, which can affect data consistency, understandability and accuracy during data collection, analysis and utilisation.

In Table 3, 45.7% (*n* = 48) of the EHPs indicated to have never participated in the review of data indicators in the data collection tools, whereas 34.3% (*n* = 36) indicated rarely. Seven of the 11 municipalities provided evidence to suggest that their EHPs and managers have participated in the review of data collection tools, which was last performed in the year 2020, led by the Provincial Department of Health. This indicates that the indicators in the data collection tools are due for review. The results indicate that with the last review, there was less participation from those at an operational level. With less participation of the EHPs in the review of indicators, the relevance, understandability and relatability of the data to be collected and reported can be affected.

On data quality dimensions, starting with data timeliness, evidence was found to suggest that data were submitted for further reporting within the accepted deadlines in most cases. Records of the reviewed documents indicated consistent timeous submissions in 10 municipalities. The submission of data or reports before the accepted deadlines contributes to the timeliness of the data, which improves its quality and on-time utilisation.

In terms of accuracy and reliability, the data from the completed data collection tools were tallied with data collation tools completed mostly by managers in 10 municipalities. The prescribed methodologies in standard operating procedures, guidelines, legislation, etc., were reported to be regularly followed by most respondents (*n* = 69; 65.7%) when they collected data, as indicated in Table 3. Data verification was reported to be conducted regularly by 34.3% (*n* = 36) of the EHPs, indicating inconsistency. Furthermore, evidence of data verification and validation was found through document reviews in only six municipalities. Considering this evidence and that from EHPs, these results demonstrate poor data verification and validation among the studied municipalities. This also raises concerns about the execution of the managers’ role in ensuring data quality. The Environmental Health Standard Operating Procedure on Information Management states that operational managers must verify all the data before onward submission (15). Data verification is important for promoting accountability, completeness of data, accuracy, consistency, reliability and integrity (56).

### 3.6 Data management support

Data management support was found to be lacking for most EHPs. As shown in Table 3, few EHPs reported receiving support regularly (*n* = 10; 9.5%). The results further revealed that those who received data management support were supported mainly by their line managers (*n* = 49; 46.6%), quality assurance officials (*n* = 36; 34.2%) and informatics officials (*n* = 19; 18%). However, data management support did not ease the work burden from data collection on the EHPs (*p* *=* 0.372). Document reviews in the 11 municipalities that were studied, two municipalities had in-house auxiliary services in the form of quality assurance and informatics sections, which supported operational practitioners and their offices on data management. Moreover, the provincial Department of Health Data Management Unit and Environmental Health Directorate also provided oversight support to all the municipalities. Sixty-one-point nine per cent (*n* = 65) of the EHPs indicated the lack of administrative support concerning environmental health information as part of the challenges regarding data management and quality issues.

### 3.7 Training of EHPs on data management and information utilisation

On the training of EHPs on data and information management, it was found that the focus in the studied municipalities was more on data collection and capturing in the data input forms (48.5%; *n* = 51), as well as on data collation (22.8%; *n* = 24). Few respondents reported having been trained in data analysis (*n* = 14; 13.3%), data use (*n* = 9; 8.5%), and information management, interpretation and presentation (*n* = 10; 9.5%). Forty-four (41.9%) respondents reported having never been trained. This indicates a gap in instilling purpose-driven data collection among EHPs and information utilisation for planning and decision-making in the delivery of EHS.

### 3.8 Data analysis

The results shown in Table 3 revealed that 45.7% (*n* = 48) of the EHPs regularly engaged in data analysis to interpret collected data in their settings. Even though a few (*n* = 14; 13.3%) EHPs were trained in data analysis. Statistical tests revealed that the manner in which the EHPs reported their engagement in data analysis was not influenced by their years of experience (*p* *=* 0.120), level of computer literacy (*p* *=* 0.437) and digital literacy (*p* *=* 0.284). Interestingly, they were also not discouraged by the work burden they perceived from data collection (*p* *=* 0.496), as well as the existence of the duplication of information during parallel reporting (*p* *=* 0.524). The results also indicate that when indicators were discussed, the respondents understood their data better and engaged in data analysis (*p* *<* 0.001; Rho=0.363). This significant moderate positive correlation indicates that discussing indicators enhances EHPs’ understanding of data and promotes engagement in data analysis.

As part of data analysis, the document reviews revealed that eight municipalities had evidence of data analysis in the form of reports. However, with respect to data visualisation, only three municipalities displayed and visualised their analysed data in dashboards, charts, graphs, infographics and maps. The data extracted from the municipalities in this regard indicated the ability of the Web-DHIS software to record and store aggregated data for the municipalities and conduct data analysis, visualisation and extraction of reports. Six municipalities provided evidence indicating the utilisation of Web-DHIS to analyse and derive reports. These findings suggest that Web-DHIS capabilities were not fully utilised in the other municipalities. Furthermore, 81% (*n* = 85) of the EHPs did not have access to this software. This suggests that EHPs mainly use manual methods to analyse data. The results further revealed that access to Web-DHIS did not have an effect (*p* *=* 0.456) on EHPs’ engagement in data analysis. Given the lack of a vigorous digital data management system, data are summarised rather than technically analysed to probe trends and patterns, predict risk and public health emergencies, and provide states of environmental health conditions in communities to enable effective decision-making in terms of interventions.

### 3.9 Data feedback

Document reviews suggested that data feedback was provided to EHPs in seven municipalities. The EHPs indicated receiving data feedback from their supervisors in meetings (*n* = 73; 69.5%), via email (*n* = 52; 49.5%), verbally (*n* = 47; 44.7%) and through written reports (*n* = 10; 9.5%). Other EHPs (*n* = 18; 17.1%) indicated that they did not receive data feedback in this regard. Data feedback was mentioned by 46.6% (*n* = 49) of the EHPs as part of the areas that needed to be strengthened. These results from the EHPs suggest a lack of uniformity in the provision of data feedback by the supervisors in the studied municipalities. The Standard Operating Procedure on Information Management for EHS directs supervisors to provide data feedback to EHPs on a monthly and quarterly basis through standardised reports that should form part of the information and management meetings for service and programme management (15).

At all levels of the health system, data feedback is important. In this study, on data feedback from the Provincial Department of Health (Provincial and District Health Offices) to the municipalities, evidence was found in four municipalities. However, it was found that the Environmental Health Directorate within the Provincial Department of Health facilitates a provincial informatics committee to discuss environmental health data matters quarterly. All municipalities that provide EHS are represented in this committee. The formation of this committee is premised on the requirements of the District Health Management Information System Policy, which dictates that the Provincial Department of Health provide feedback to its districts and improve data quality and programme performance (14). However, apart from this committee, the Policy also requires other feedback mechanisms (e.g., email and telephone communications, site visits, symposiums, etc.) to be done to discuss any relevant matters to optimise data management and strengthen the health system. However, evidence to suggest these discussions in operational meetings was found in the minutes of previous meetings in only seven municipalities. The other four municipalities failed to produce minutes of previously held meetings with these discussions. Therefore, data feedback was less optimal at the operational and district (municipal) levels.

### 3.10 Information utilisation

The results shown in Table 3 demonstrate the low existence of a culture of information utilisation among the majority of the EHPs. A total of 45.7% (*n* = 48) of the EHPs indicated that they use data to inform, plan and make decisions in their routine work, in a frequency that is sometimes and below. A moderate positive correlation was found between EHPs’ engagement in data analysis and the use of data-driven insights for planning and decision-making (*p* *<* 0.001; Rho = 0.504), while a weaker but significant positive correlation was observed for data integration (*p* *<* 0.001; Rho = 0.323). This suggest that EHPs who actively analysed and integrated data were more likely to use information to inform decisions and planning in their routine work. The results of this study further indicate that the way the EHPs participated in data utilisation was not influenced by their level of education (*p* *=* 0.699), years of experience (*p* *=* 0.366), level of satisfaction with the existing EHIS (*p* *=* 0.619) or the duplication of information during reporting (*p* *=* 0.259). However, the work burden from data collection negatively affected the use of data for planning and decision-making by the EHPs (*p* *=* 0.038) at a small scale (Rho=−0.203).

Evidence indicating the availability and reporting of data to senior management was found in 10 municipalities. Notably, this happens through reports for service delivery budgets and implementation plans, annual performance plans and operational plans. In one municipality, it was indicated that they also provide data through reports to higher administrative levels, but no evidence was produced. Furthermore, through the review of documents in the municipalities, reports were provided in five municipalities to demonstrate the utilisation of data for planning and decision-making. Six municipalities provided evidence suggesting that they engage in the utilisation of data for monitoring and evaluation. The reviewed records also revealed that in five municipalities, there were data-driven discussions in management meetings, and there were related matters that were referred to higher administrative levels internally for executive interventions. However, few data elements were noted as subjects of discussion. Notably, the indicators in the existing system are not comprehensive enough to advance planning and decision-making, as they are based on aggregated data. This makes the system more quantitative than qualitative, lacking the granularity needed to make localised decisions. With respect to strategic planning, five municipalities had strategic plans that were backed up by environmental health data on environmental health-related issues. At an operational level, six municipalities presented plans that included environmental health data as the basis for outlined interventions, which is not a good reflection. With respect to the evaluation of the delivery of EHS and employee performance, eight municipalities provided records to suggest the use of data as a measuring tool.

Although some municipalities showed evidence of the utilisation of data for planning and decision-making, inconsistency was noted. This is further supported by the results from the EHPs on their sentiments on the utilisation of data on the DHIS/EHIS for planning and decision-making by their supervisors and senior managers. As shown in Table 3, most of the EHPs (*n* = 79; 75.2%) indicated that their supervisors use data on a frequency of sometimes and below. Therefore, most EHPs were not satisfied with the use of data by their supervisors.

The results of this study also indicate that most of the EHPs did not see enough evidence in their settings to indicate that their senior managers were using data effectively in planning and decision-making. This finding is based on the fact that 15.2% (*n* = 16) of the EHPs indicated that their senior managers use data in planning and decision-making on a frequency that is above sometimes, as shown in Table 3. The comparison of these results with the EHPs’ level of satisfaction with the existing information system on how it addresses environmental health matters indicated a positive moderate association (*p* *<* 0.001; Rho=0.320). This implies that when the satisfaction of the EHPs with the existing EHIS increased, their sentiment toward information utilisation by senior management also increased, and vice versa. This provides that the satisfaction of the respondents with the EHIS is linked with the relevance and use of the data provided by the same system to back plans and make impactful decisions at a senior management level, and vice versa. Therefore, when an EHIS provides comprehensive data, senior management becomes in a better position to use that data for planning and making better decisions, which may then satisfy practitioners as data collectors and users.

## 4 Discussion

### 4.1 Data collection and reporting

As the conceptual framework for this study indicates, data collection, capture and reporting are the first steps in the data flow process, and they influence the performance of an RHIS at a later stage during information utilisation (26, 36). In this study, the source documents for the environmental health data and the data collection tools (weekly and monthly data input forms) were found to be mainly paper-based. This corroborates the findings of the study, which highlighted the prevalence of paper-based tools in South Africa's RHISs (27). In that study, the use of paper-based systems was found to put the quality of data at risk of being compromised (27). Especially since paper-based tools and manual processes increase the chances of data errors during collection and capture (4). The results of this study show that the studied EHIS is mainly paper-based at an operational level since data are captured only on the Web-DHIS once they are collated for the entire municipality. This finding is similar to that of a study in which the malaria reporting system within the DHIS was evaluated and found to be paper-based at the data collection level and electronically at the collation and reporting stage (28). While this hybrid approach facilitates data aggregation and reporting, it limits the real-time availability of information. The capture of data once a month into the Web-DHIS means that emerging environmental health threats may not be early detected and communicated promptly, thereby delaying public health responses.

The delayed availability of data can affect the timely provision of environmental health interventions where they are needed, leading to a reactive approach instead of proactively protecting human health and the environment. A study conducted in Uganda revealed that paper-based systems have bureaucratic delays that affect the data transmission rate, availability and timeliness (29). The Web-DHIS is structured in an organisational hierarchy from the national to the local level to capture data; its last organisational unit is a local municipality (57). It does not allow data to be captured at the subdistrict and ward levels within a municipality. As a result, monthly data input forms from subdistrict managers are collated for the whole municipality for consolidated data to be captured into the Web-DHIS. If Web-DHIS allows data to be captured at subdistrict and ward levels, it could improve data availability, timeliness and utilisation for quick responses to environmental health issues at an operational level. The weekly and monthly data input forms used for data capture are made up of the Environmental Health National and Provincial Indicators Datasets. These indicators are not comprehensive in terms of the scope of work of the EHPs in the provision of EHS. These findings confirm the statement by Wright and Street, who wrote that the use of a mainly paper-based data management system that falls short in terms of addressing comprehensive environmental health issues is a challenge in South Africa for public health surveillance (18). Indicators need to respond to data demands and information needs that are based on the scope of work and the issues at hand (38).

The data collection tools must also be designed to be user-friendly for implementation at the service rendering level (55). The design of these tools is crucial as a mechanism for eliminating duplications and promoting the quality of data from the onset (58). In this study, in nine municipalities, the duplication of data was noted in their weekly and monthly data input forms. This finding supports the duplication of data reported by the majority of the EHPs (*n* = 93; 88.6%), which influenced their ability to observe the need for the removal of some elements (*p* *=* 0.004; Phi = 0.316). This moderate positive association found between the need for the removal of some data elements and experiencing work and data duplication in reporting, indicate that duplication is an important factor contributing to perceptions of excessive or unnecessary data collection. This finding reflects inefficiencies in the information management system, where similar information is collected or reported multiple times, increasing workload without adding value. However, another finding revealed that the data collection tools were not comprehensive enough, as 88.6% (*n* = 93) of the EHPs recommended the addition of other data elements. Sixty-seven-point percent (*n* = 71) of the EHPs revealed to be below moderately satisfied with the existing EHIS. Therefore, a comprehensive review is required to address redundancy, streamline reporting requirements and promote its reliability, completeness, and relevance of the information generated.

In a study that evaluated the reproductive health information system in Tshwane, South Africa, it was suggested that to improve its performance and promote its usability, some irrelevant data elements had to be eliminated, and the use of paper-based tools was recommended to be discontinued (40). In the present study, the data in the EHIS within the DHIS were too aggregated and lacked the granularity to advance planning and decision-making for the development of targeted interventions. Owing to this gap left by the Department of Health's EHIS, parallel reporting takes place when municipalities internally and other external stakeholders require more localised and comprehensive data. This leads to the development of new templates from time to time to generate the required data for reporting. This then causes an administrative burden to the EHPs (*n* = 66; 62.8%) for repeated data compilation and reporting. Therefore, the existing EHIS within the DHIS does not provide enough data to meet the information or data needs required for other reports. These findings support previous research that identified a lack of data-driven approaches in EHS, as well as a need for a comprehensive EHIS in South Africa (18, 21, 22). This calls for drastic changes in the management of environmental health data and information utilisation. In New Zealand, the adoption of a digital information system streamlined data management processes and delivered environmental health intelligence by enabling the detection of risks early and providing warnings and response actions to stakeholders and citizens (19).

In this study, the results revealed limited access to tools for trade (*n* = 18; 17.1%) and smart devices (*n* = 16; 15.2%), highlighting a dire need for transformation and the injection of resources. The effectiveness of EHPs heavily depends on their access to appropriate and advanced tools, equipment, and resources necessary for monitoring health risks, automating manual processes and developing relevant interventions in time (16, 23–25). Without these tools, the ability to protect public health and the environment can be severely compromised. These findings support assertions that technologically advanced data management systems and technical tools are required in environmental health, from data collection to the real-time submission of data and easy generation of reports at just the press of a button (22, 24).

Streamlined data management systems are associated with positive user perceptions and contribute to the successful implementation of RHISs (59). In this study, the EHPs valued the data they submitted to the EHIS, recognising both its importance (*n* = 99; 94%) and usefulness (*n* = 93; 89%). Furthermore, a moderate positive correlation was observed between data as important and perceiving it as useful (*p* *<* 0.001; Rho=0.503), suggesting that appreciation of data value is closely linked to its utilisation. Despite these positive perceptions, most EHPs (*n* = 79; 75%) reported negative perceptions regarding data collection. This was associated with the user-friendliness of the data collection tools, with a moderate negative correlation indicating that lower user-friendliness was linked to a greater perceived work burden (*p* *<* 0.001; Rho=−0.357). In a review paper by Tull, it was reported that the duplication of data and work overburdens health practitioners and then affects their effectiveness in their line of duty, as well as their ability to produce high-quality data (60). Similarly, another study reported that practitioners who perceived data management as an extra burden were less likely to utilise information generated from the DHIS effectively (61). Owing to the high workload, data capturing may not be performed with the necessary care, resulting in data collection errors, thereby compromising data quality (36, 40). In the present study, weak but statistically significant positive correlations were found between perceived data collection burden and both EHPs age (*p* *=* 0.012; Rho=0.243) and years of experience (*p* *=* 0.026); Rho = 0.217). These findings suggest that older and more experienced EHPs were slightly more likely to perceive data collection as burdensome; however, the small effect sizes indicate limited influence. This highlights the lack of meaningful changes and advancements in tools and the system over time. The work of EHPs is mainly data-based to monitor environmental factors that may have detrimental effects on human health and the environment in the community, and to develop interventions (62). Therefore, the existence of a burden among the EHPs demonstrates the need to review the whole system to streamline data collection processes, improve operational efficiency and support effective information management.

### 4.2 Data quality, storage and security

High-quality data are needed for better information, informed decision-making, and improved public health (58). This means that data quality is one of the important factors in the successful implementation of an RHIS to generate reliable information (63, 64). The data generated by the studied EHIS in the municipalities that participated were found to be of fair quality. This is because the dimensions of data timeliness, accuracy, completeness, reliability and integrity were found, even though the system was mainly paper-based. However, this level of data quality remains suboptimal and indicates the existence of systemic loopholes that need to be addressed for data quality improvement. Data that are of good quality should be complete, accurate, relevant, current and up-to-date, consistent, reliable, of integrity, understandable, available, secured and accessible for utilisation (40, 64). While data quality is often viewed as a technical issue, it is also influenced by governance, organisational capacity, and resource allocation (36). The continued reliance on paper-based processes reflects broader institutional constraints, including limited investment in digital infrastructure, inadequate information management capacity, and competing service delivery priorities.

Evidence from the document review process suggests that in 10 municipalities, weekly and monthly input forms were completed in full and submitted within accepted deadlines. The methodologies prescribed in the legislation, standard operating procedures and guidelines were consistently followed by most EHPs (*n* = 79; 75.2%). Therefore, the above-outlined results highlight several important data quality dimensions, such as accuracy, consistency and reliability. They further demonstrate a degree of organisational compliance and commitment to routine information management. However, the inability of the existing data collection tools to comprehensively capture the scope of EHS, as outlined in the Environmental Health National Norms and Standards, points to a governance challenge relating to indicator design and system responsiveness. This affects the scope and relevance of generated data to environmental health challenges in the community. Hence, parallel systems were noted to be developed to source further information when there are environmental health issues of concern, suggesting that the current EHIS may not adequately meet operational information needs. This finding corroborates that the review of the system and its indicators is overdue and required to enhance relevance. This fragmentation can undermine data standardisation, increase workload, and weaken the completeness of information available for decision-making.

The finding that 45.7% (*n* = 48) of the EHPs had never been involved in indicator review processes further highlights potential governance and organisational capacity gaps. The exclusion of EHPs who are operational and collect data may have downstream effects on data quality and utilisation at a later stage due to poor data understandability and relatability. This may also reduce the relevance, usability, and ownership of indicators by the EHPs. This concern is reinforced by the absence of evidence in six municipalities that did not have evidence to support their participation in these reviews, which were last conducted in the year 2020. The District Health Management Information System Policy requires indicators to be reviewed every two years to ensure that it is up-to-date and aligned with the priorities of the health system (14). Strengthening participatory governance mechanisms and creating structured opportunities for operational EHPs involvement in indicator development and review may improve the alignment of the EHIS with operational realities and emerging environmental health priorities.

In terms of data availability, 81% (*n* = 85) of the EHPs did not have access to the data in the web-DHIS for analysis, visualisation and generation of reports. This finding suggests that information access is not solely a technical issue but also reflects organisational and governance arrangements relating to user permissions, decentralised decision-making, and information-sharing practices. Limited access to Web-DHIS, as well as paper-based storage of work records in piled-up cabinets and storerooms, and the retrieval of historic data are noted as challenges and may restrict opportunities for local evidence-based planning and weaken the information-use culture that is essential for RHIS performance. As a result, this study has shown that because of paper-based processes, some data quality dimensions are lacking, and interventions are needed to improve the EHIS and its quality. These dimensions include data availability and its relevance to current environmental health and public health issues. A study on data quality in TB control reported that data quality issues were more prevalent in paper-based records than in digital records (65). Another study in Ghana reported that paper-based data collection resulted in more mistakes and less accurate data being entered into the system (66). Therefore, improving data quality requires not only the adoption of digital tools and systems but also organisational reforms that empower EHPs as active users of information.

One of the most notable findings emerging from this study, as mentioned above, is that the existing EHIS is mainly paper-based. The use of digital technologies, from data collection to the storage of work records and data, was very low. A lack of tangible record management procedures (including archiving), backup plans, and non-uniformity in this regard was also found, indicating broader institutional weaknesses in information governance. This finding corresponds with the findings of the study, where in one of the largest municipalities in South Africa, inefficient paper-based record-keeping practices and a lack of standardisation were found in the management of environmental health data and information from food premises (22). As a result, it is concluded that the existing EHIS system harbours risks of damage and data loss, as well as degradation over time. This system is further vulnerable to data manipulation due to insecure storage and a lack of access control measures, which can affect the protection of sensitive information, the integrity of data, and data quality in general. These findings suggest that the use of paper-based storage of records and data threatens the safety and security of data and further fragments the centralisation of data for easy accessibility and sharing. This finding agrees with Bimerew's findings, which showed that the lack of a digital record storage system in mental health information management affected data quality, confidentiality and the safety of patients’ information (67). In that study by Bimerew, document reviews revealed data storage challenges, as they also observed piled-up documents on the floor and tables in a fragmented manner (67). Therefore, digitalisation can be a solution to these data storage and safety challenges by adopting technologically advanced security measures and data backup infrastructure to safeguard the data. In response to data and record management issues in the provision of EHS, Mokoatle also highlighted the need for a technologically assisted system as a solution (22). Another study by Kgolane suggested that environmental health data and information management need to be improved and enhanced to safeguard effective record keeping and evidence management, and adopt a standard digital centralised information system (24). While digitalisation presents a potential solution, successful implementation will require sustained investment, leadership commitment, workforce capacity development, standardisation of record management practices and clear governance structures to support digital transformation. Without these enabling conditions, the benefits of digital technologies may not be fully realised.

### 4.3 Quality assurance, data feedback and data management support

Quality assurance checks include various activities that seek to enhance the value of data by improving its quality, and that of the work conducted (58). In the process of improving data quality, supervision and regular data management support are also important for addressing data quality threats and operational challenges for data collectors (68). The conceptual framework for this study maps quality assurance and data management as important pillars in the entire data flow process. However, the findings suggest weaknesses in these support systems, as elements of poor-quality assurance and inconsistent data management support were evident. This is due to the lack of discussions on indicators, inconsistent data verification and validation by managers, and the possibility of human errors in the use of paper-based tools. Discussions on indicators enable a common understanding and interpretation of the data among practitioners, and data verification and validation can promote data quality (15, 69). Therefore, there is a need for quality assurance and data management support improvement. These challenges affect data quality directly, and further point inadequate management oversight, insufficient institutionalisation of quality assurance practices, and uneven investment in information management capacity across municipalities.

The lack of administrative support (*n* = 65; 61.9%) and competent staff in data management (*n* = 44; 41.9%) further suggests capacity constraints within the organisational structures responsible for EHS. Notably, receiving data management support from supervisors was not associated with a reduced work burden (*p* *=* 0.372), indicating that existing support mechanisms may be insufficient and lack the technical depth required to address operational challenges effectively. This finding highlights a gap for stronger organisational capacity, whereby dedicated auxiliary support sections within EHS provide technical support where it is needed, and ensure that there are established accountability structures and managerial competencies that extend beyond routine supervision to include data quality improvement and system strengthening. In this study, it was found that just two municipalities had auxiliary sections on quality assurance and informatics, and they were better off in terms of their capacity for environmental health data and information management, suggesting that investment in specialised support functions may contribute to improved system performance. These findings highlight the importance of these support services in data management and EHS generally to identify and address areas of concern for implementing quality control protocols and standards to ensure continuous improvement. From a policy perspective, this points to the need for formalising quality assurance and health informatics functions within EHS, supported by adequate funding and human resources. This can further open an opportunity for the enrolment of data feedback, as part of support and quality assurance.

According to the PRISM framework, data feedback is one of the important processes in the functioning of an RHIS (36). This provides an opportunity for data-driven discussions between practitioners who collect and report data and those who receive reports. Based on the results in Section 3.9, it is evident that data feedback was less optimal and inconsistent with the provisions of the Standard Operating Procedure on Information Management for EHS. Data feedback discussions must be conducted monthly and quarterly on priority indicators and data quality (15). Effective data feedback is important to provide a platform for engagements to address identified challenges, provide necessary solutions, and share lessons learned, as well as best practices towards maximum utilisation of information to protect public health (69–73). Data feedback discussions can also be incorporated into capacity-building programmes to bring alignment and run purposeful sessions. Strengthening routine feedback processes could therefore enhance both data quality and information utilisation while fostering a stronger culture of evidence-based decision-making.

As part of data management support, capacity-building is also one of the important aspects for continuous improvement. In this study, a lack of training of the EHPs on data analysis (*n* = 14; 13.3%), interpretation and presentation (*n* = 10; 9.5%), and utilisation for planning and decision-making (*n* = 9; 8.5%) reflected broader workforce development needs within environmental health information management. Training programmes can improve individual competencies and provide an opportunity for discussions on indicators/elements. The positive moderate association between discussions on indicators and engagement in data analysis (*p* *<* 0.001; Rho = 0.363) suggests that opportunities for learning and engagement can strengthen users’ ability to understand and apply data effectively. This reinforces the importance of institutionalising ongoing training, mentorship, discussions and indicator review processes as part of routine information management practice. In a study on reproductive health information management, insufficient orientation, training, and a lack of these discussions led to 64.9% of health practitioners not being aware of newly updated definitions of indicators and data elements of their RHIS (40). Another study that assessed information utilisation in a high-HIV-prevalence setting revealed that practitioners’ poor understanding of the definitions of indicators and elements in their data tools contributed to the poor recording of data and led to data quality issues (30). Capacity-building programmes provide a platform for discussions on emerging public health threats, changes in legislative frameworks, clarification of roles and responsibilities, operational challenges, data quality and other data management matters (61, 74). Hence, it is evident that capacity building in data management is key to ensuring data quality, and also enhancing the knowledge and proficiency of the users on the system and its utilisation to maximise benefits (74).

### 4.4 Utilisation of environmental health information for planning and decision-making

The utilisation of routine health information at all levels of the health system is essential for planning and decision-making to ensure effective and efficient service delivery to the community (61, 75). The utilisation of routine health information is key for health institutions to be better run and deliver the best outcomes (76). The conceptual framework for this study also alludes to this fact assertion. Hence, in the development of operational and strategic plans, insights that have been derived from routine health data must be included in these documents to show their relevance and responsiveness to issues of public health concern to be addressed. In routine work at an operational level as well as at the strategic level, decisions should be based on data-driven insights.

At the operational level of the provision of EHS, this study revealed that the culture of information utilisation existed among almost half of the EHPs (*n* = 48; 45.7%). This reflects a positive foundation for evidence-informed practice. However, this culture appears to be constrained by systemic challenges that include limited access to technical tools and smart handheld devices, which results in reliance on manual data management processes. These constrains likely contribute to increase workload and inefficiencies, as the study found statistically significant but weak negative correlation between work burden from data collection and data utilisation (*p* *=* 0.038; Rho=−0.203). This suggests that as the burden of data collection increases, the use of information for planning and decision-making tends to decrease. Even though the relationship is weak, it highlights an important operational dynamic, whereby there is excessive demand on data collection that may reduce the time, motivation and capacity of EHPs to engage in higher-level data utilisation. This finding underscores a potential imbalance within the information system, where greater emphasis is placed on data generation rather than data utilisation. In this context, data collection risks becoming a routine administrative task, rather than a meaningful input into planning and decision-making. Addressing this imbalance requires investments in digital tools, process automation, and workflow optimisation, which can reduce the burden on EHPs and enable a stronger focus on data utilisation. Strengthening this aspect is critical for improving the overall effectiveness of the EHIS and ensuring that collected data translates into actionable insights. In a study that was conducted in Uganda, it was also found that the shortage of working resources and redundant administrative processes overburdened health workers, which negatively affected data quality and utilisation (77). Among the EHPs in this study, as indicated in 3.10, significant relationships were found between the utilisation of information and their engagement in data analysis (*p* *<* 0.001), as well as data integration (*p* *<* 0.001). This indicates that the ability to analyse and combine data from different sources plays a critical role in enabling meaningful information utilisation. This means that EHPs who are more actively engaged in analysing data and integrating datasets are more likely to translate raw data into actionable insights, thereby strengthening evidence-informed planning and decision-making at the operational level. This finding supports the call for the power of data from an RHIS to be first demonstrated where it is produced (78). It also emphasises the importance of strengthening analytical capacity within EHS.

With respect to middle management in the studied municipalities, the utilisation of environmental health information was moderate. Although EHS generates a substantial volumes of data, relatively few elements were discussed in meetings and incorporated into strategic plans and escalated executive structures for high-level intervention. This may reflect a broader governance and system design challenges rather than a lack of information utilisation alone. At the executive level, environmental health information utilisation was found to be minimal. Owing to the inability of the existing EHIS to generate sufficiently comprehensive and strategic information to clearly demonstrate the scope, impact, and value of EHS to senior decision-makers. Consequently, environmental health issues may receive limited visibility within organisational planning and resource allocation processes.

## 5 Limitations of the study and suggestions for future research

In this study, the use of a self-administered questionnaire to collect data from EHPs limited the findings of this study to the understanding and interpretation of the respondents on the concepts that were used in the questions. The document reviews could not be conducted in all EHS subdistricts or remote offices within the municipalities due to their vastness. However, the structure of the data extraction sheet targeted documents and processes that were available and accessible in the central or head offices of the EHS departments in the municipalities. Not all EHS rendering municipalities participated in this study, as 2 did not agree to form part. A 10% margin of error was also used in the sample size calculation, which is relatively wide and reduces the precision of the estimates. As a result, the generalisability of the findings of this study is limited. Furthermore, the convenience sampling approach of EHPs further restricts generalisability, as this also limits the external validity of the results. The possibility of volunteer bias is acknowledged, as EHPs who chose to participate may have different perspectives and responses from those who did not participate. Respondents may differ from non-respondents in factors that are relevant to the study such as digital literacy, internet access, workload, and engagement in information management practices. This may have influenced the findings, with a possible overrepresentation of more digitally engaged respondents and underrepresentation of those facing greater constraints, or vice versa. As much as the entire population was invited to strengthen coverage and the document review was conducted to augment the survey results, the potential for non-response bias limits the representativeness of the results. Moreover, due to the use of a uniform online survey link, as well as the questionnaire's structure and anonymity, the researcher could not provide a breakdown of the distribution of respondents across the municipalities. Data was also collected at a single specific time, limiting the ability to observe changes over time. While the study provides useful baseline insights, future studies with larger samples, narrower margins of error and defined outcome variables to enable robust multivariable modelling are recommended to improve precision and external validity. The focus of future research is recommended to extensively assess data quality, explore digitalisation, and expand the assessment of environmental health information management to the whole of South Africa.

## 6 Conclusion

This study provides empirical evidence on the state of environmental health information management in eleven South African municipalities, using the conceptual framework in Figure 1. The assessment focused on the EHIS embedded within the DHIS, and associated processes from data collection to information utilisation. It was found that the existing EHIS and the data management processes that were utilised in the studied municipalities are largely paper-based, leading to implications on data quality and safety, as well as data fragmentation which inhibits the centralisation of data for easy accessibility and sharing. This study showed that the existing EHIS in the studied municipalities did not provide enough information for utilisation in planning and decision-making. The study further revealed that weaknesses in quality assurance, data feedback, and data management support are influenced not only by technical shortcomings but also by broader governance, organisational capacity, and resource constraints. Inconsistent indicator reviews, limited involvement of frontline EHPs in system improvement processes, inadequate access to data, shortages of technical and administrative support, and insufficient investment in health informatics functions were identified as key barriers to information system performance. These findings highlight the need for stronger governance arrangements, dedicated quality assurance and informatics structures, enhanced managerial oversight, and sustained capacity development to improve environmental health information management. Despite these challenges, the study found encouraging evidence of a growing culture of information utilisation among the majority of the EHPs, showing enthusiasm and motivation for the power of data in the provision of EHS. Information utilisation was positively associated with data analysis and data integration, demonstrating the importance of analytical capacity in translating routine data into evidence for planning and decision-making. However, information utilisation remained constrained by data collection burdens, limited digital tools, and an information system design that appears to prioritise data production over data utilisation. Furthermore, environmental health information was found to have limited visibility at higher levels of management, reducing its influence on strategic planning, resource allocation, and policy decisions. Overall, the findings suggest that strengthening environmental health information management requires a comprehensive systems approach that extends beyond technological improvements alone. In the short -term, priority actions should include maximising existing technologies within municipalities to kickstart the digital transition; reviewing and rationalising indicators and reporting requirements; expanding EHPs access to data via the WebDHIS; strengthening quality assurance, support and feedback mechanisms; and implementing targeted capacity-building programmes. Equally important is the institutionalisation of governance structures that promote accountability, data quality and evidence-based planning and decision-making. In the medium-term, sustainable financing mechanisms should be pursued through budgeting processes, public-private partnerships and government digital transformation grants to support modernisation and digitalisation of environmental health information management. In addition, the development of a digital EHIS should be entrenched within the relevant legislation, policies and environmental health related strategic frameworks to ensure long-term commitment, accountability and implementation. As a custodian for health services and health information in South Africa, the National Department of Health should lead this transformation towards a centralised, integrated, and comprehensive digital EHIS that serve as a single source of truth for environmental health data and intelligence. These reforms are essential to improve the quality, relevance, accessibility and utilisation of environmental health information, strengthen public health surveillance and response capacity, and enhance the delivery of EHS to reduce the environmental health burden of disease and yield better health outcomes in communities.

## Data Availability

The original contributions presented in the study are included in the article/Supplementary Material, further inquiries can be directed to the corresponding author.
