# Supplementary material for: From data collection to action: environmental health information management practices in eleven municipalities in the east coast of South Africa
Source: Front Health Serv. 2026 Aug 18;6:1890673. doi: 10.3389/frhs.2026.1890673 (PMC13529946; doi:10.3389/frhs.2026.1890673)
Supplement: Supplementary file 2 [file Table2.docx]

**SELF-ADMINISTERED QUESTIONNAIRE FOR ENVIRONMENTAL HEALTH PRACTITIONERS**

*Questionnaire for Environmental Health Practitioners (EHPs) at an operational level.*

**Please mark the answer with a cross (X) and be advised that you are allowed to tick more than 1 answer where applicable.**

**SECTION A: DEMOGRAPHIC DATA**

1. Gender

| Male | 1 |
| --- | --- |
| Female | 2 |
| Other | 3 |

2. Age group

| 20 to 30 years | 1 |
| --- | --- |
| 31 to 40 years | 2 |
| 41 to 50 years | 3 |
| 51 to 60 years | 4 |
| Above 61 years | 5 |

3. Highest educational qualification obtained

| Diploma or National Diploma | 1 |
| --- | --- |
| Bachelor’s Degree, Bachelor of Technology Degree or National Higher Diploma | 2 |
| Honours, Post-Graduate Diploma or Professional Bachelor’s Degree | 3 |
| Master’s Degree | 4 |
| Doctoral Degree | 5 |

4. Are you registered with HPCSA as an independent practitioner?

| Yes | 1 |
| --- | --- |
| No | 2 |

5. Years of experience as an Environmental Health Practitioner

| Less than 5 years | 1 |
| --- | --- |
| 6 to 10 years | 2 |
| 11 to 20 years | 3 |
| 21 years and above | 4 |

6. Area of employment

| District Municipality | 1 |
| --- | --- |
| Metropolitan Municipality | 2 |
| Local Municipality | 3 |

|  | **Basic** | **Intermediate** | **Advanced** |
| --- | --- | --- | --- |
| 7. Level of computer literacy (knowledge and ability to use computers and related technologies) | 1 | 2 | 3 |
| 8. Level of technological digital literacy (Proficiency in using digital devices (smartphones, laptops, tablets) and digital platforms (internet, social media etc.) | 1 | 2 | 3 |

**SECTION B: TRAINING OF EHPS AND THEIR PERCEPTIONS ON THE EHIS**

9. What training have you received on data management, District Health Information System (DHIS) or Environmental Health Information System (EHIS)? (Cross (X) all answers that apply)

| Weekly/monthly data collection tools (data input forms) | 1 |
| --- | --- |
| Data collation (compilation) | 2 |
| Data analysis | 3 |
| Data use for planning and decision-making | 4 |
| Information management, interpretation and presentation | 5 |
| Never been trained | 6 |
| Other, specify_______________________ | 7 |

10. To what extent does data collection add to the work burden in your work duties?

| To no extent | 1 |
| --- | --- |
| Small extent | 2 |
| Moderate (average) extent | 3 |
| Large extent | 4 |
| Very large extent | 5 |

11. How would you rate the importance of the data that you feed to the EHIS?

| Not important | 1 |
| --- | --- |
| Slightly important | 2 |
| Moderately important (average) | 3 |
| Important | 4 |
| Very important | 5 |

12. How would you rate the usefulness of the data that you feed to the EHIS?

| Not useful | 1 |
| --- | --- |
| Slightly useful | 2 |
| Moderately useful (average) | 3 |
| Useful | 4 |
| Very useful | 5 |

**SECTION C: QUALITY ASSURANCE AND DATA MANAGEMENT SUPPORT**

|  | **Never** | **Rarely** | **Sometimes** | **Often** | **Always** |
| --- | --- | --- | --- | --- | --- |
| 13. In your institution, how often do you discuss definitions of indicators or elements on the data collection tools (data inputs forms) that you use? | 1 | 2 | 3 | 4 | 5 |
| 14. How often does your supervisor or line manager conduct verification of the data that you feed onto the data collection tools? | 1 | 2 | 3 | 4 | 5 |
| 15. As an EHP in your institution, how often do you receive support on data collection and management? | 1 | 2 | 3 | 4 | 5 |
| 16. How often do you participate in the review of data elements or indicators in the data collection tools? | 1 | 2 | 3 | 4 | 5 |

17. Who provides support in your institution in relation to health information management? (Cross (X) all answers that apply)

| Monitoring and Evaluation or Quality Assurance Official(s) | 1 |
| --- | --- |
| Supervisor | 2 |
| Informatics Official(s) | 3 |
| No one | 4 |
| Other, specify__________ | 5 |

**SECTION D: DATA COLLECTION AND STORAGE**

18. What are the sources of the data that you feed to the data collection tools (data input forms) for reporting in your routine work? Cross (X) all answers that apply

| Records of daily/weekly activity books | 1 |
| --- | --- |
| Notes in a diary or notebook | 2 |
| Inspection checklists | 3 |
| Inspection reports | 4 |
| Measurements results from technical instruments (sound level meter, air quality detector etc.) | 5 |
| Other(s), specify: ____________________________ | 6 |

19. To what extent are the data collection tools used in your institution user-friendly?

| Not user-friendly | 1 |
| --- | --- |
| Slightly user-friendly | 2 |
| Moderately user-friendly (average) | 3 |
| User-friendly | 4 |
| Very user-friendly | 5 |

20. To what extent do you use prescribed methodologies (in the legislation, Standard Operating Procedures, Guidelines etc.) when collecting data?

| Never | 1 |
| --- | --- |
| Rarely | 2 |
| Sometimes | 3 |
| Often | 4 |
| Always | 5 |

|  | **Yes** | **No** |
| --- | --- | --- |
| 21. Do you experience or notice any duplication of work and data, during the generation of data or when completing data collection tools? | 1 | 2 |
| 22. Do you think more environmental health elements/indicators should be added to the data collection tools? | 1 | 2 |
| 23. Are there elements/indicators that you think should be removed from the data collection tools? | 1 | 2 |

24. In your own opinion, what is your level of satisfaction with how the existing EHIS cover or address current Environmental Health issues?

| Not at all satisfied | 1 |
| --- | --- |
| Slightly satisfied | 2 |
| Moderately satisfied (average) | 3 |
| Satisfied | 4 |
| Very satisfied | 5 |

25. Please cross the working tools that are at your disposal to be able to conduct data collection and reporting when executing your duties as an EHP. Cross (X) all answers that apply.

| Pens and pencils | 1 |
| --- | --- |
| Paper | 2 |
| Computer | 3 |
| Hand-held devices (e.g. tablets, smartphones etc.) | 4 |
| Internet | 5 |
| Printers | 6 |
| Scanners | 7 |
| Cameras | 8 |
| Technical instruments (sound level meter, air quality detector etc.) | 9 |
| Other(s), specify: __________________ | 10 |

26. How is the data and the records of the work that has been completed stored in your workplace (i.e. inspection reports, compliance notices issued, complaints registers, etc.)? Cross (X) all answers that apply.

| Manually - Paper-based system (e.g. files, registers, books etc.) | 1 |
| --- | --- |
| Computer-based system (e.g. Share-drive and computer programs) | 2 |
| Intermediate or advanced web-based system (e.g. DHIS2) | 3 |
| Software (i.e. Application useable in hand-held devices and computers) | 4 |
| Cloud storage (Online – e.g. One Drive, Google Drive etc.) | 5 |
| Other, specify: ______________________________________ | 6 |

|  | **Never** | **Rarely** | **Sometimes** | **Often** | **Always** |
| --- | --- | --- | --- | --- | --- |
| 27. How often do you experience the duplication of information in terms of reporting, where similar data/information is required by different offices (internally) and external stakeholders or departments (parallel reporting**)?** | 1 | 2 | 3 | 4 | 5 |
| 28. How often do you participate in the collation (compilation) of data in your office or department? | 1 | 2 | 3 | 4 | 5 |

**SECTION E: DATA ANALYSIS AND PROVISION OF FEEDBACK**

29. How often do you analyse the data that you collect?

| Never | 1 |
| --- | --- |
| Rarely | 2 |
| Sometimes | 3 |
| Often | 4 |
| Always | 5 |

30. Do you have access to the Web DHIS?

| Yes | 1 |
| --- | --- |
| No | 2 |

31. How do you receive feedback from your supervisor on the data that you submit to him/her? Cross (X) all answers that apply

| Verbally (including telephonic) | 1 |
| --- | --- |
| Emails | 2 |
| Meetings | 3 |
| Written feedback reports | 4 |
| No feedback received | 5 |
| Other(s), please specify: _________ | 6 |

**SECTION F: COLLABORATIONS WITH OTHER STAKEHOLDERS AND DATA INTEGRATION**

|  | **Never** | **Rarely** | **Sometimes** | **Often** | **Always** |
| --- | --- | --- | --- | --- | --- |
| 32. As an EHP, individually, how often do you consider data from other databases/sources outside Environmental Health (e.g. Data from clinics, Business Licensing, NICD, South African Air Quality Index etc.) in your work to conduct risk assessments and apply Environmental Health interventions? | 1 | 2 | 3 | 4 | 5 |
| 33. How often do you work with other stakeholders or service departments (water authorities, business licensing, Primary Health Care etc.) for joint interventions (integration of services), to address issues identified through data sharing and integration? | 1 | 2 | 3 | 4 | 5 |
| 34. How often do you collaborate with academic or research institutions to use environmental health data for research purposes? | 1 | 2 | 3 | 4 | 5 |

**SECTION G: DATA UTILISATION FOR PLANNING AND DECISION-MAKING**

|  | **Never** | **Rarely** | **Sometimes** | **Often** | **Always** |
| --- | --- | --- | --- | --- | --- |
| 35. How often do you use your collected data to inform, plan and make decisions in your work? | 1 | 2 | 3 | 4 | 5 |
| 36. How often do you think your supervisor or line manager utilises the data provided on the DHIS/EHIS for planning and decision-making? | 1 | 2 | 3 | 4 | 5 |
| 37. How often do you think the senior management of your department utilises the data provided on the DHIS/EHIS for planning and decision-making? | 1 | 2 | 3 | 4 | 5 |

**SECTION H: PROSPECTS OF DIGITALISATION**

38. How satisfied are you with developments or advancements in your organisation towards the digitalisation of working systems and improving EHIS to enhance the provision of Environmental Health Services, and embrace the Fourth Industrial Revolution?

| Not satisfied at all | 1 |
| --- | --- |
| Slightly satisfied | 2 |
| Moderately satisfied (average) | 3 |
| Satisfied | 4 |
| Very satisfied | 5 |

39. What are the challenges or concerns that you have regarding data management, flow and quality in your institution? Cross (X) all answers that apply.

| Lack of administrative support in relation to Environmental Health Information | 1 |
| --- | --- |
| Lack of human resources | 2 |
| Lack of financial and material resources to do the work | 3 |
| Lack of understanding of data and information collected | 4 |
| Lack of feedback on data and information submitted | 5 |
| Lack of necessary technical equipment to collect data during technical assessments | 6 |
| Lack of trained and competent staff on data management | 7 |
| Lack of commitment from the management of the department | 8 |
| Lack of commitment from the executive of the municipality | 9 |
| Other, please specify:______________________ | 10 |

40. In your opinion, what should be done to improve EHIS in your area of work, institution, province or country-wide?

________________________________________________________________________________________________________________________________________________________________________________________________________________________________________________________________________________________________________________________

**THANK YOU FOR YOUR PARTICIPATION**
