# Supplementary material for: From data collection to action: environmental health information management practices in eleven municipalities in the east coast of South Africa
Source: Front Health Serv. 2026 Aug 18;6:1890673. doi: 10.3389/frhs.2026.1890673 (PMC13529946; doi:10.3389/frhs.2026.1890673)
Supplement: Supplementary file 3 [file Table3.docx]

**DATA EXTRACTION SHEET**

| **Date completed** |  | | | |
| --- | --- | --- | --- | --- |
| **Unique identification code** |  | | | |
| **Documents to be scrutinized** | **Themes and targeted information for data collection and analysis** | **Data extraction questions** | **Yes** | **No** |
| Policies, Guidelines and Standard Operating Procedures. | 1. Data flow process. | 1A. Is there a policy, guideline or SOP on EHIS/DHIS or information management in general? | 1 | 2 |
|  |  | 1B. Is the data flow process outlined? | 1 | 2 |
|  | 2. Roles and responsibilities of different users. | 2A. Are the roles and responsibilities of different users outlined? | 1 | 2 |
|  | 3. Information management practices and procedures. | 3A. Are information management practices and procedures employed in the municipality outlined? | 1 | 2 |
| Working documents for data collection and collation (i.e. source documents, environmental health data collection tools, registers etc.). | 4. Duplication of data collection | 4A. Is there any duplication of data collection on the working documents? | 1 | 2 |
|  | 5. Completeness of data collection tools | 5A. Are the data collection tools completed in full? | 1 | 2 |
|  | 6. Relevance of data collection tools | 6A. Do the data collection tools cover the data to be collected by Environmental Health Services, comprehensively? (Are the elements or indicators in the data collection tools in line with the EH NNS) | 1 | 2 |
|  |  | 6B. Do the data collection tools ask about environmental health interventions implemented to address non-compliance or any public health issues? | 1 | 2 |
|  |  | 6C. Is there any evidence to suggest that data collection tools get reviewed to ensure that they remain relevant to the current times and Public Health issues | 1 | 2 |
|  | 7. Data storage | 7A. Does the studied municipality have a document management system/policy to address data storage? | 1 | 2 |
|  |  | 7B. Is the storage of records as source documents access controlled? | 1 | 2 |
|  | 8. Data timeliness | 8A. Are the data collection tools submitted within the accepted deadlines? | 1 | 2 |
|  | 9. Data accuracy and reliability | 9A. Does the data recorded in completed data collection tools (data input forms), tally with the data recorded in the data collation tools completed by managers? | 1 | 2 |
|  |  | 9B. Is there any evidence of data verification or validation conducted by managers? | 1 | 2 |
| Reports. | 10. Collation, analysis and reporting of collected data. | 10A. Is there any evidence in reports that shows that collected data gets collated and analysed? | 1 | 2 |
|  |  | 10B. Are there any displays or visualisation of data to show that data has been analysed and transformed into information? | 1 | 2 |
|  |  | 10C. Is there any evidence that shows that analysed data/information is reported further to other offices or departments? | 1 | 2 |
|  |  | 10D. Are the reports submitted to external stakeholders within the accepted deadlines? | 1 | 2 |
|  | 11. Provision of data feedback. | 11A. Is there any evidence in reports that shows that data feedback is given to those who collect data (EHPs)? | 1 | 2 |
|  |  | 11B. Is there any evidence on feedback reports from external stakeholders that data analysis reports are sent to? | 1 | 2 |
|  | 12. Utilisation of data for planning and decision-making. | 12A. In reports, is there any information that shows that previously reported data is used for planning and decision-making? | 1 | 2 |
|  |  | 12B. Is the information or data accessible in the DHIS, to advance planning and decision-making? | 1 | 2 |
|  |  | 12C. Is there any evidence that suggests that data is used for monitoring and evaluation? | 1 | 2 |
|  |  | 12D. Are there any data-driven matters that have been referred to higher or executive level for interventions? | 1 | 2 |
| Minutes of meetings held. | 13. Provision of feedback and discussions on information management, as well as performance and service delivery gauges based on the reported data. | 13A. Is there any evidence in the minutes of previous meetings that shows that data feedback is provided? | 1 | 2 |
|  |  | 13B. Are there any data-driven discussions for planning and decision-making? | 1 | 2 |
|  |  | 13C. Are there any discussions on employees’ performance, using data as a measuring tool? | 1 | 2 |
|  |  | 13D. Is there any evidence to show that data is used to measure the provision of Environmental Health Services? | 1 | 2 |
| Plans or draft plans (operational and strategic plans). | 14. Utilisation of data for planning and decision-making | 14A. Are there any operational plans in the municipality or department that are backed up by environmental health data? | 1 | 2 |
|  |  | 14B. Are there any strategic plans in the municipality or department that are backed up by environmental health data? | 1 | 2 |
|  | 15. Prospects of digitalisation of services and embracing the Fourth Industrial Revolution. | 15A. Are there any plans in the municipality that speak to digitalisation and embracing the Fourth Industrial Revolution? | 1 | 2 |
| Additional notes |  | | | |
