# Supplementary material for: From data collection to action: environmental health information management practices in eleven municipalities in the east coast of South Africa
Source: Front Health Serv. 2026 Aug 18;6:1890673. doi: 10.3389/frhs.2026.1890673 (PMC13529946; doi:10.3389/frhs.2026.1890673)
Supplement: Supplementary file 4 [file Table1.docx]

**Supplementary material 1: Epi Info Version 7.2.6.0 sample size calculation for EHPs**
